# RANet: a custom CNN model and quanvolutional neural network for the automated detection of rheumatoid arthritis in hand thermal images

**DOI:** 10.1038/s41598-023-42111-3

**Published:** 2023-09-20

**Authors:** R. K. Ahalya, Fadiyah M. Almutairi, U. Snekhalatha, Varun Dhanraj, Shabnam M. Aslam

**Affiliations:** 1 Department of Biomedical Engineering, College of Engineering and Technology, SRM Institute of Science and Technology, Kattankulathur, Chennai, Tamil Nadu 603203 India; 2 Department of Biomedical Engineering, Easwari Engineering college, Ramapuram, Chennai, Tamil Nadu India; 3 Department of Information Systems, College of Computer and Information Sciences, Majmaah University, 11952 Al Majmaah, Saudi Arabia; 4 Department of Physics and Astronomy, University of Waterloo, Waterloo, ON Canada; 5 Department of Information Technology, College of Computer and Information Sciences, Majmaah University, 11952 Al Majmaah, Saudi Arabia

**Keywords:** Diseases, Health care, Rheumatology, Engineering, Mathematics and computing, Nanoscience and technology, Optics and photonics

## Abstract

Rheumatoid arthritis is an autoimmune disease which affects the small joints. Early prediction of RA is necessary for the treatment and management of the disease. The current work presents a deep learning and quantum computing-based automated diagnostic approach for RA in hand thermal imaging. The study’s goals are (i) to develop a custom RANet model and compare its performance with the pretrained models and quanvolutional neural network (QNN) to distinguish between the healthy subjects and RA patients, (ii) To validate the performance of the custom model using feature selection method and classification using machine learning (ML) classifiers. The present study developed a custom RANet model and employed pre-trained models such as ResNet101V2, InceptionResNetV2, and DenseNet201 to classify the RA patients and normal subjects. The deep features extracted from the RA Net model are fed into the ML classifiers after the feature selection process. The RANet model, RA Net+ SVM, and QNN model produced an accuracy of 95%, 97% and 93.33% respectively in the classification of healthy groups and RA patients. The developed RANet and QNN models based on thermal imaging could be employed as an accurate automated diagnostic tool to differentiate between the RA and control groups.

## Introduction

Rheumatoid arthritis (RA) is a degenerative and incendiary disease of the joints that weakens bone and cartilage, leading to disability^1,2^. It is an auto-immune disease that affects the small and large joints if left untreated^3^. Most of the RA patients experience the symptoms such as extreme fatigue, inflammation, joint space narrowing, and bone erosion. Globally, the prevalence of RA was approximately 20 million in 2019. In India, 0.75% (around 10 million) of people are affected by RA^4–6^. The early detection of RA is crucial to avoid risk factors such as autoantibodies and joint degeneration^7^. RA affects the women three times more than men^8^.

The primary clinical features of RA are associated with inflammatory cytokine release in the synovial tissue and aberrant synovial cell proliferation in the joint. The main cell type implicated in the pathogenesis of RA is fibroblast-like synoviocytes (FLS), which play a vital role in the synovium's hyperplasia and the development of the vascular pannus^9^. FLS involves in subsequent deterioration of bone and cartilage in the joint. In RA, the body's immune system might be prompted to attack specific body tissues, and its root cause is unknown. The affected joints become worse as a result of this circumstance. The aggravation may eventually cause damage to the ligament, the joint and adjacent bone fragments. The main issue in diagnosing RA is subclinical inflammation, a condition in which clinicians are unable to identify the inflammation^10^. The various diagnostic methods that detect the subclinical inflammation are C-reactive protein (CRP), Erythrocyte Sedimentation Rate (ESR), radiograph, ultrasonography, and MRI^11^.

Radiography is considered as a standard method for detecting RA; however, scoring radiographs requires more time to review per patient^12^. The predominant changes in the small joints caused due to RA are also challenging to assess through radiographs. Ultrasonography and magnetic resonance imaging (MRI) are the other modalities used to detect RA^13^. Imaging using ultrasonography is user-dependent, and MRI is more expensive. These modalities could visualize only the structural changes and cannot visualize the temperature changes in the tissues caused due to RA. Thermal imaging has the ability to visualize the temperature changes caused due to the synovial inflammation in RA^14^.

Thermograms diagnose local and systemic inflammatory changes caused by superficial dermal microcirculation in affected hand regions^14^. The synovitis incendiary caused due to the RA is associated with elevated skin temperature in the joints. Thermal imaging is a non-invasive modality with less computation cost than other modalities^15^. Therefore, thermal imaging could be employed as a pre-screening tool for diagnosing RA. Several studies have shown a momentous variation in the temperature values of RA and healthy subjects^16–19^.

Artificial intelligence (AI) techniques have been used for medical image classification tasks^20–23^. AI-assisted detection of RA using hand thermal images will make it easier for physicians to identify RA. Morales et al. assessed inflammation caused by RA in hand thermograms using machine learning (ML) classifiers^24^. The authors employed thermographic joint inflammation score (ThermoJIS) from the hand thermal images for classification. They extracted the features using scale-invariant and rotation-invariant algorithms. A *k*-NN model was employed as the ML classifier to detect RA. The authors obtained a classification accuracy of 79% to evaluate RA. The authors suggested that the thermoJIS technique would help physicians to identify synovitis. Their study utilized hand-crafted feature extraction techniques, which is a tedious task. The present study employed an automated feature extraction method by incorporating pre-trained and custom models for the classification of RA.

Another study by^25^ proposed an evaluation of RA using joint temperature, demographic, and clinical scores from the hand thermal images. The authors identified the RA patients with mild, moderate, and severe disease activity based on the intensity heat maps. The temperature characteristics from hand thermal images and wrists of RA patients and normal participants were studied by Gatt et al.^18^. The authors used a logistic regression model to categorize control and RA groups. The authors obtained a significant temperature difference of 2.03 °C and 3.06 °C in the palm and finger region respectively for the normal subjects and RA patients. Their study proved that the thermal imaging could be used as a pre-screening method to evaluate RA. Their study has limited assessment parameter like temperature measurements, for the evaluation of RA.

Several studies on automated detection of hand thermograms were based on ML algorithms^26–28^ Majority of the studies have used temperature values as the input to the ML classifier. Limited literature used convolution neural network (CNN) models to classify control subjects and RA patients in the hand thermal images. Hand-crafted feature extraction is a tedious procedure performed in ML classification, whereas automated feature extraction was carried out by deep learning (DL) models. The millions of images are used in the ImageNet dataset to train the pre-trained models for classification^29^. These models include many layers and are computationally expensive for diagnosing RA. Therefore, a customized RANet model was developed in the current study for diagnosing RA to overcome these constraints.

Recent advancements in quantum computing have an extensive impact on medical applications^30–32^. The hybrid algorithms that integrate quantum and classical CNNs are gaining significant importance. One such hybrid method is quanvolutional neural network (QNN), which combines quantum computing with conventional CNN^33^. It modifies the traditional CNN by adding a new transformation layer called a quanvolutional or quantum convolutional layer. These layers can extract the features from the input image by applying spatial transformations to the image's sub-regions using variational or random circuits. This work evaluates the performance of the QNN model with the custom RA Net model for RA categorization. The objectives of the present study are described as follows: -

- To build pre-trained and customized models for predicting and classifying the control and RA groups.
- To validate the performance of the custom model features using a feature selection method and classification using ML classifiers.
- To compare the performance of the custom RA Net model and QNN models in the classification of healthy and RA subjects.

The major contributions of the current study include:

- The custom RANet was developed for the classification of RA patients and the healthy groups.
- A QNN model was constructed for the application of RA and compared with the RANet model.
- A hybrid model was built to validate the performance of RANet model by extracting deep features from the model and to perform the classification using various ML classifiers.

The organization of the paper is described as follows: The first section demonstrates the introduction of RA and its prevalence, literature review related to RA in thermal imaging, introduction to QNN and objectives of the proposed study. The detailed methods related to data collection, thermal imaging acquisition protocol, modified pre-trained model, developed RANet, QNN, ML classification using deep RA Net features are elaborated in section two. In the third section, the results illustrating the performance metrics of ML and DL classifiers, QNN classification are described. The discussion and conclusions of the study were explained in the last section.

## Materials and methods

### Subjects

For the proposed study, real-time thermograms were acquired from SRM Medical College, Hospital and Research Centre (SRM MCH&RC), Kattankulathur, Tamil Nadu. Patients who were confirmed with RA by an expert rheumatologist were included in the study. The Institute Ethics Committee (Human Studies) of SRM MCH&RC approved the study with IEC Number: 2449/IEC/2021. The guidelines and regulations of the present study were performed in accordance with Declaration of Helsinki. The thermal images were acquired for the out patients visited the Department of Rheumatology, SRM MCH&RC from 13 September 2021 to 6 January 2022. According to the consensus report given by the Indian Rheumatology Association (IRA), the participants were divided into two groups: normal (N = 50) and RA (N = 50)^34^. The subjects who are participated in the study signed an informed consent form. Subjects with diabetes mellitus, who have undergone recent physiotherapy, hypertension, and fever, were excluded from the study.

### Protocol for thermal imaging

**Figure 1:**
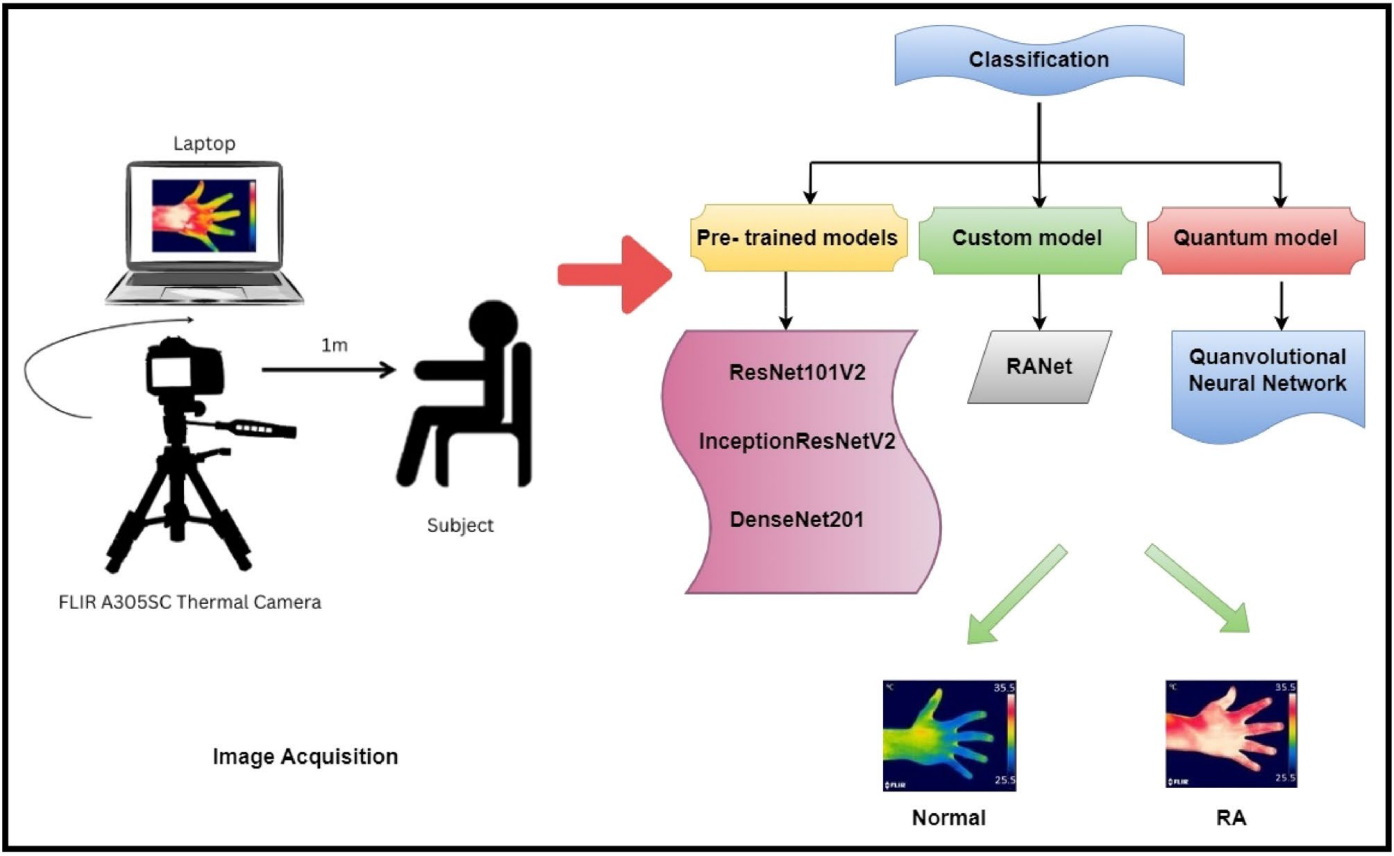
Experimental setup of the study for the classification of RA.

Patients were advised to remove the ornaments before undergoing a thermal imaging procedure. They were seated in a temperature-controlled (20 °C) dark room with both hands exposed for 15 min^35^. For the proposed study, FLIR (Forward Looking Infrared) A305SC thermal camera was used to acquire hand thermal images. The thermal camera was focused at a 1 m distance from the subject's hand. Thermal images of the right hand and left hand of dorsal, ventral, AP view were acquired for the proposed work. FLIR camera has a supporting in -built software tool to store, analyze and process the hand thermograms. A rainbow color palette was chosen in this proposed work to detect the hot spot regions in the hand thermograms. The temperature values of the finger joints were measured using a square tool of size 10 × 10 mm from the FLIR software. Figure 1 demonstrates the study's experimental setup for the RA classification.

### Dataset splitting for thermal images

The study comprised of healthy participants (N = 50) and RA patients (N = 50) to categorize the RA and healthy groups. A total of 600 hand thermal images (3 views from each hand; 3 views × 2 hands × 50 normal images = 300; similarly, 300 images for RA Patients) were recorded using a FLIR A305SC thermal camera. Total 600 thermal images were split into 80–20%, in which 480 images were used for training and 120 images for testing. The training 480 images were increased to 1440 images using data augmentation techniques such as elastic deformation with a standard deviation of 2, brightness, and scaling of 10%. Additionally, a 70–30% split was used for the CNN models' training (1008 images) and validation (432 images).

### Modified pre-trained CNN models based on the transfer learning technique

Humans could master intricate procedures in one field and apply that knowledge to complete related activities in another^36^. Likewise, transfer learning in the context of deep learning used previously trained knowledge for other tasks^37,38^. The ImageNet dataset contains thousands of real-world images to train the pre-trained CNN models. Due to the lack of massive medical imaging datasets, pre-trained models trained on natural images were employed in medical image analysis^39^. The transfer learning technique resolves this issue by transferring the learned parameters from the ImageNet dataset and trains the pre-trained models based on their weights for the classification of various diseases. Convolutional and pooling layers of pre-trained models are modified, while fully connected layers are trained from scratch using the medical imaging dataset. The fully linked layers were fine-tuned by adding four dense layers to predict the healthy and RA subjects. The current study utilized modified pre-trained models such as ResNet101V2, InceptionResNetV2, and DenseNet201 for the classification of RA.

**Figure 2:**
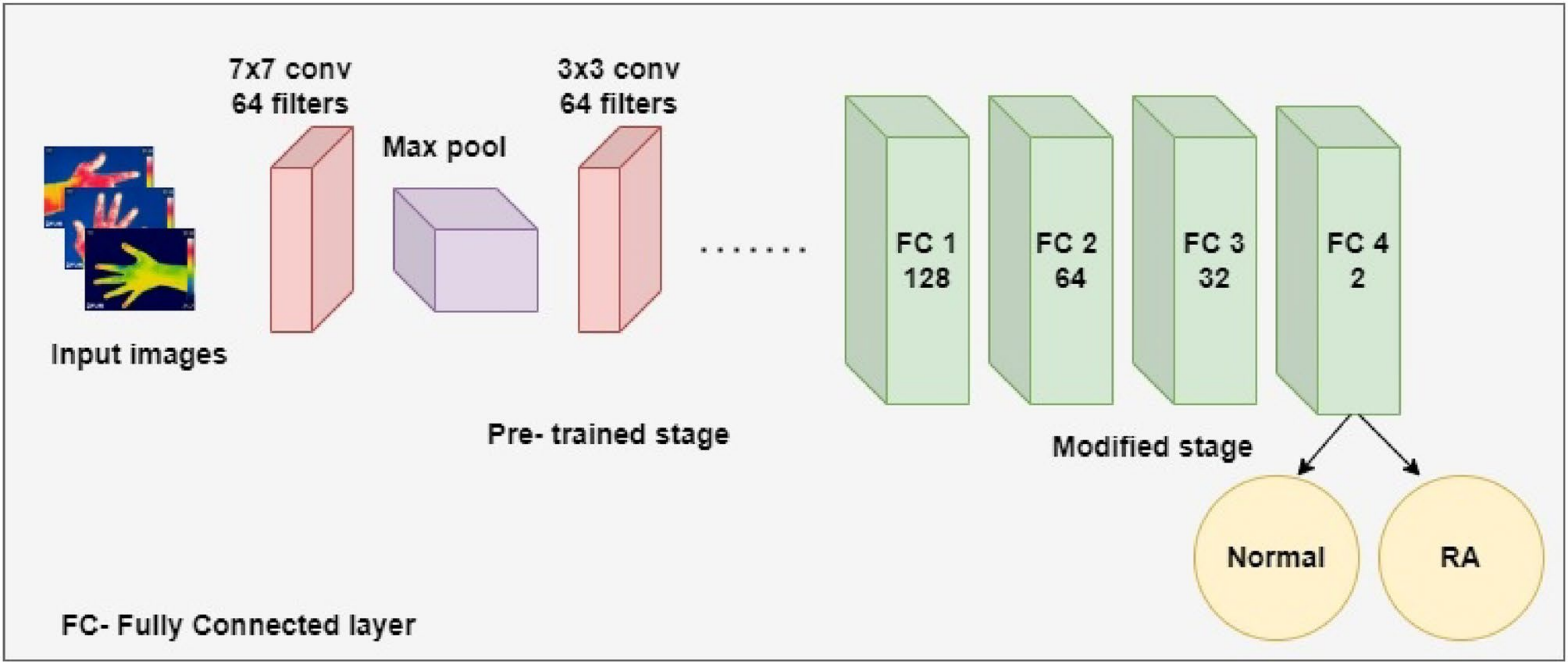
Architecture diagram of ResNet101V2 for the classification of RA.

ResNet101V2 is the modified version of the deep residual net (ResNet), which is comprised of 101 deep convolutional neural layers trained on thousands of images in the ImageNet dataset^40^. ResNet architecture used skip connections which connect the activation function, ReLU of a layer to further layers by skipping the other layers. It is stacked with several residual units, and the units are represented as^41^,

1 $${\text{W}}_{{\text{q}}} = {{\{ \text{W}}}_{{\text{q, k}}} |1 \le {\text{k}} \le {\text{K}}\}$$

2 $${\text{Y}}_{{\text{q}}} = {\text{ h(x}}_{{\text{q}}} {) } + {\text{ f(x}}_{{\text{q}}} ,\;{\text{w}}_{{\text{q}}} {)}$$

3 $${\text{x}}_{{{\text{q}} + 1}} = {\text{ f(y}}_{{\text{p}}} {)}$$

where x_q_ is the input and x_q+1_ is the output of the qth block, ‘K’ is the number of residual blocks, ‘F’ is the residual function, ‘f’ is the ReLU activation function, ‘W_q_’ represents the weight and bias of the qth residual block, h(x_q_) = x_q_ illustrates the identity mapping. The modification of ResNet101V2 is to construct a path inside a residual block and the entire architecture for feature propagation. The feature could be propagated within the entire architecture for h (x_q_) and f (y_p_) identity mappings. For deeper block ‘Q’ and shallow block ‘q’, the equation is illustrated as follows:

4 $${\text{X}}_{{\text{Q}}} = {\text{ x}}_{{\text{q}}} + \mathop \sum \limits_{{{\text{i}} = 1}}^{{{\text{Q}} - 1}} {\text{F }}\;({\text{x}}_{{\text{i}}} ,\;{\text{w}}_{{\text{i}}} )$$

where ‘X_Q_’ represents the summation of x_0_ and follows the residual function's output. The equation for backpropagation is given as:

5 $$\frac{{\partial {\text{E}}}}{{\partial {\text{xq}} }} = \frac{{\partial {\text{E}}}}{{\partial {\text{xQ}} }}\frac{{\partial {\text{xQ}}}}{{\partial {\text{xq}} }} = \frac{{\partial {\text{E}}}}{{\partial {\text{xq}} }}\left( {1 \, + \frac{\partial }{{\partial {\text{xq}} }} \mathop \sum \limits_{i = 1}^{Q - 1} {\text{F}}\;({\text{x}}_{{\text{i}}} ,\;{\text{w}}_{{\text{i}}} )} \right)$$

where ‘E’ represents the loss function. In the current study, the input hand thermal images were resized to 256 × 256 and fed into the different convolutional and pooling layers of the ResNet101V2 model. Fine-tuning of the model was performed in the last layers by adding four fully connected (FC) layers of neurons such as 128, 64, 32, and 2. Finally, the SoftMax activation function classified the output based on probabilistic values obtained from the FC layers. The architecture diagram of the ResNet101V2 for the classification of RA is illustrated in the Fig. 2.

**Figure 3:**
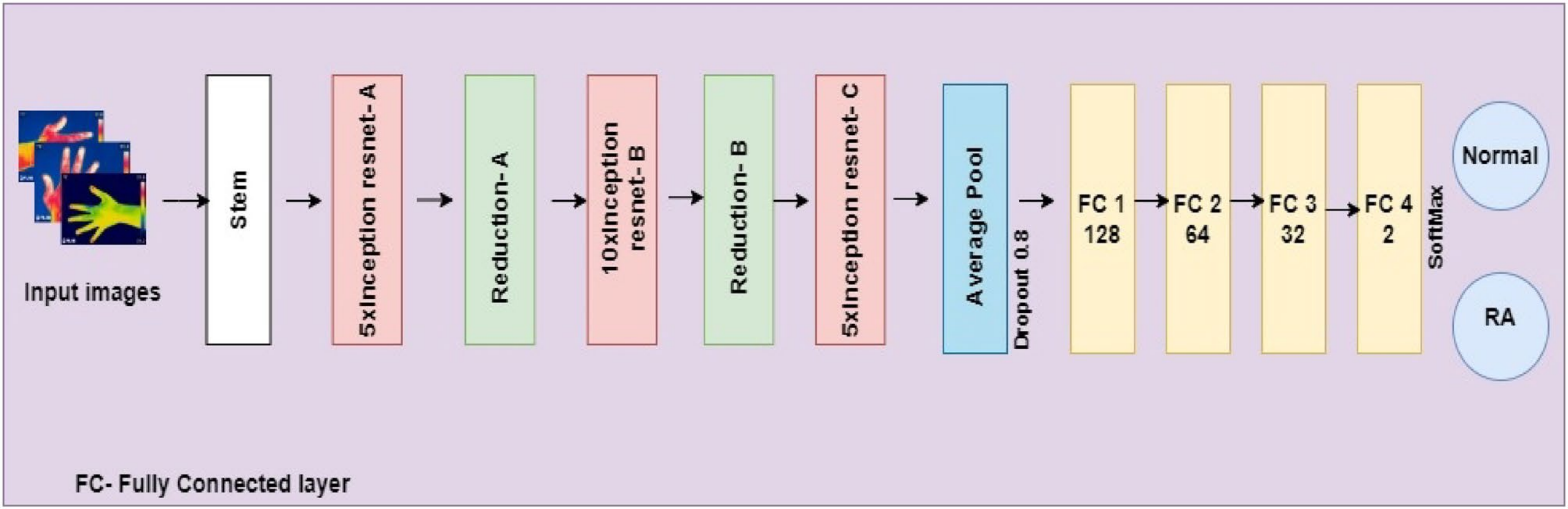
Architecture of InceptionResNetV2 model for the classification of RA.

The InceptionResNetV2 model is a combination of Inception and ResNet architectures which consists of a stem block and Inception ResNet blocks (A, B, C)^42^. Each InceptionResNet block is followed by reduction blocks (A and B), except the last InceptionResNet block C, which is preceded by an average pooling and SoftMax activation function. This architecture utilized the factorization method to decrease the kernel size, thereby reducing the overfitting problem and number of parameters. In the proposed study, the input hand thermal images were passed through all the layers of the InceptionResNetV2 model except the last layers. The final layer of the model was fine-tuned by adding four FC layers of neurons such as 128, 64, 32, and 2 neurons, and the feature vectors were fed into the SoftMax function for the estimation of RA and healthy subjects. Figure 3 depicts the Inception ResNetV2 architecture diagram to classify RA patients from healthy participants.

**Figure 4:**
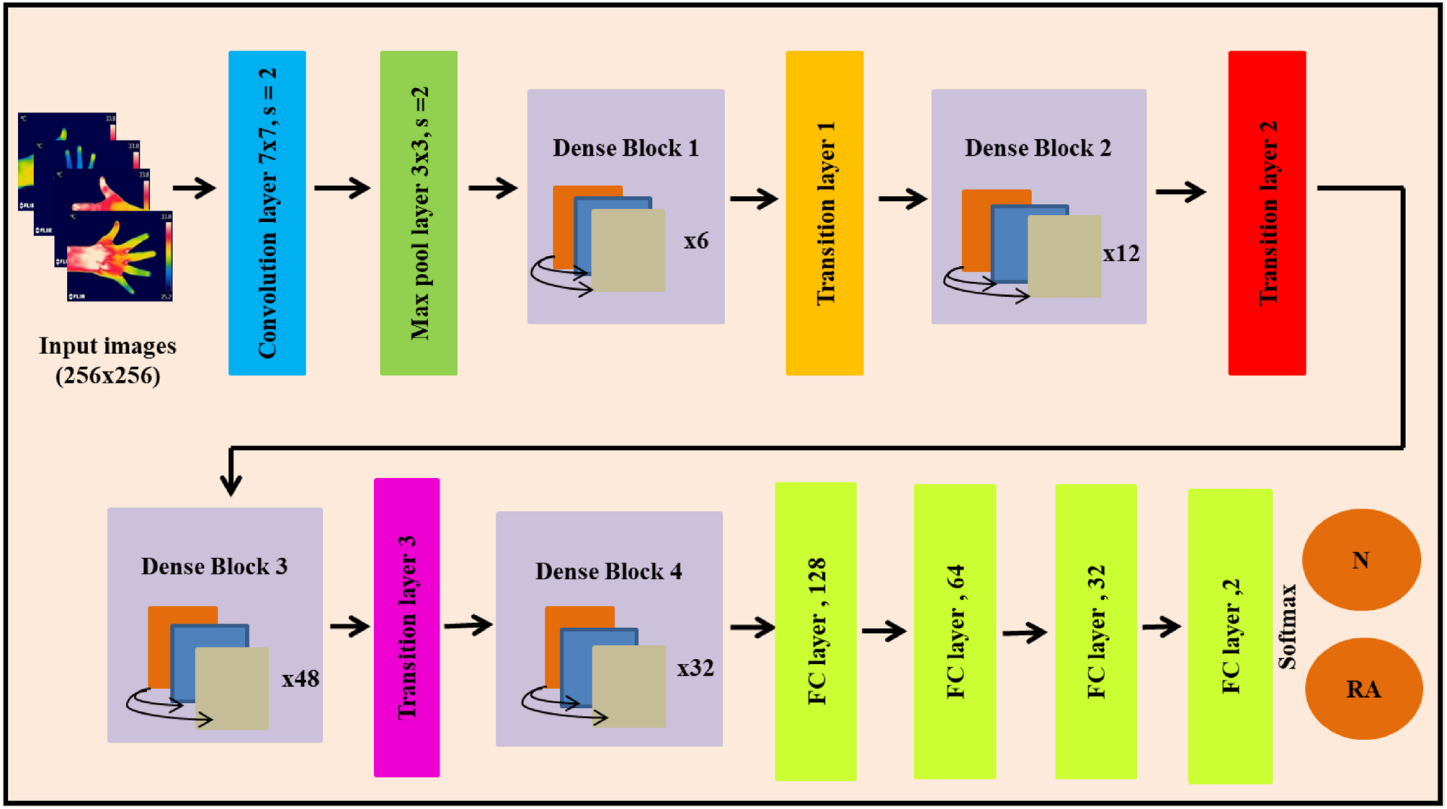
The modified architecture of DenseNet201 for the classification of RA.

DenseNet201 model comprised of 201 deep layers trained on thousands of images in the ImageNet database^43–45^. The sequential concatenation was performed instead of the concatenation of the output feature maps of the preceding layers in the Dense Net201 model. Mathematically this is illustrated as follows:

6 $${\text{X}}_{{\text{q}}} = {\text{ Hq }}\left( {\left[ {{\text{x}}_{0} ,\;{\text{x}}_{1} ,\;{\text{x}}_{2} \ldots {\text{x}}_{{{\text{q}} - 1}} } \right)} \right]$$

where x_q_ is the feature of the qth layer, ‘q’ represents the layer index, and ‘H’ is the nonlinear operation. The hand thermal images of size 256 × 265 were fed into the convolution layer with kernel size 7 × 7, stride 2, max pooling layer with kernel size 3 × 3, and stride 2. Then the features maps of the thermal images were passed to dense block 1 which consists of convolution layers with kernel sizes 1 × 1 and 3 × 3, and the layers multiplied six times within the first block. Next, these features were transferred into the transition layer 1 which comprised of a convolution layer of kernel size 1 × 1 and max pool layer of filter size 3 × 3 with stride 2. After that, these feature maps were passed to the dense block 2, which contains convolution layers of kernel sizes 1 × 1 and 3 × 3, and these layers were multiplied twelve times within the second block. Then, the next layer is the transition layer 2, which consists of convolution layer of filter size 1 × 1, an average pooling layer of filter size 2 × 2 with stride 2. These features were fed into the dense block 3 which consist of convolution layers of filter size 1 × 1 and 3 × 3, and these layers were multiplied 48 times within the third block. After that, the feature maps were fed into the transition layer 3, which contains the same layers of transition layer 2. Then the feature maps were passed into the last dense block 4, which comprised of the same convolution layers, and these layers were multiplied 32 times within the block 4. These layers are pre-trained based on the weights of the ImageNet dataset. In this study, the last FC layers comprised of thousand classes were removed, and four new FC layers were added to classify the control and RA subjects. Figure 4 represents the modified architecture of DenseNet201 for the categorization of RA and healthy subjects.

### RANet customized CNN model

The pre-trained models such as ResNet101V2, InceptionResNetV2, and DenseNet201, are trained based on the natural images in the ImageNet database. Despite altering the pre-trained models, it fails to provide reliable performance based on accuracy. Therefore, a customized CNN model (RANet) was developed to address these problems for classifying the RA and the healthy participants.

**Figure 5:**
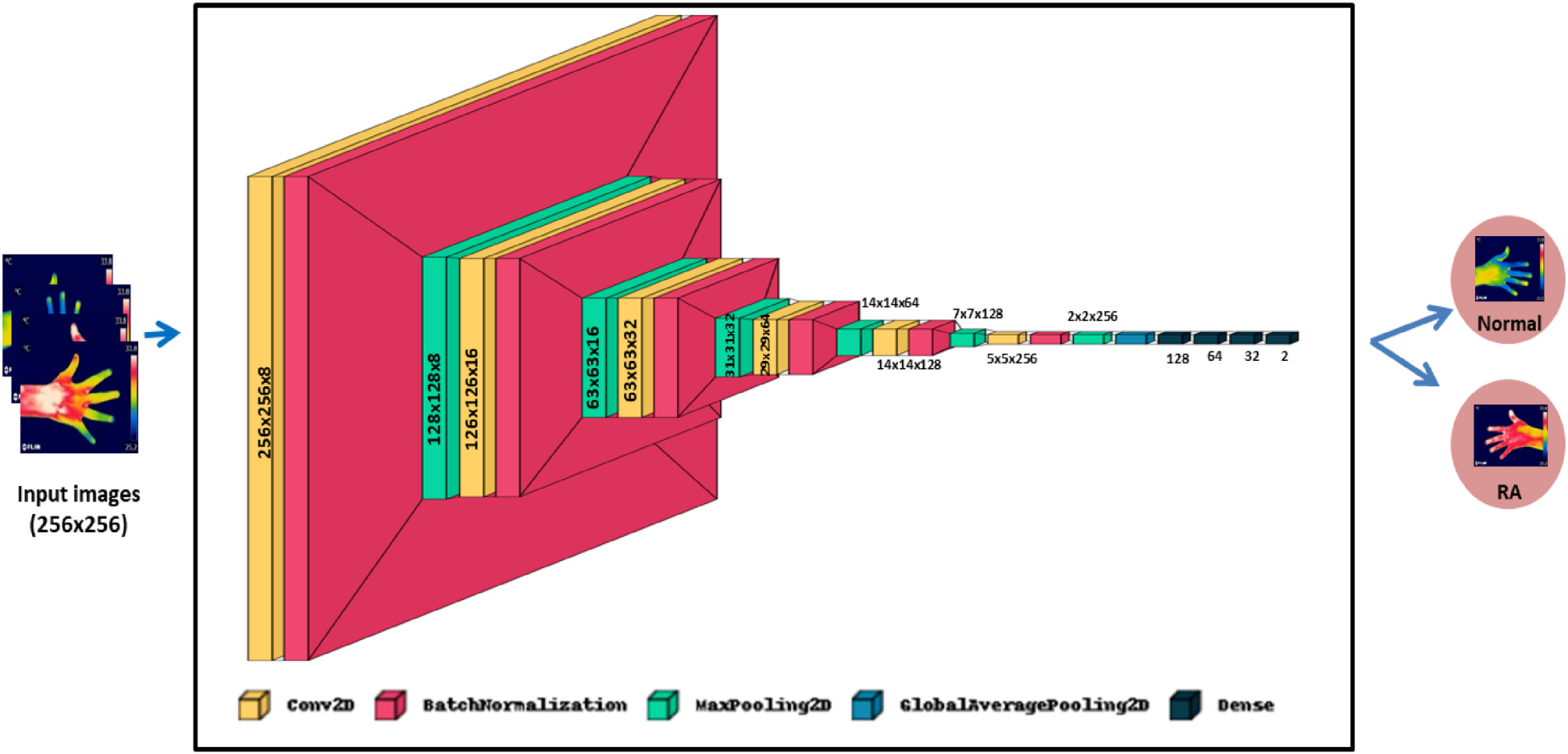
RANet model for the classification of RA.

The RANet model is the customized model for prediction of RA developed by the authors. It consists of six convolutional layers followed by max pooling, batch normalization, global average pooling layers, and four fully connected layers. The input hand thermal images of size 256 × 256 pixels were fed into a convolution layer with eight neurons, 1 × 1 kernel size, and a stride of 1. The ReLU activation function, batch normalization, and max pooling layers follow each convolution layer. Then the feature maps were then passed into a max pooling layer of kernel size 2 × 2 with stride size 2. The max pooling layers down sample the feature maps, and these features were passed into the next convolution layer with 16 neurons, 3 × 3 filter size, and stride 1. Likewise, the feature maps were passed through four convolution layers with neurons such as 32, 64, 128, and 256, and its filter sizes of 1 × 1, 3 × 3, 1 × 1, and 3 × 3 respectively. From the last max pooling layer, the feature maps were fed into the global average pooling and four fully connected layers of 128, 64, 32, and 2 neurons. Then, these feature vectors were passed to the SoftMax activation function, which classifies the control groups and RA subjects. Figure 5 represents the RANet CNN model with various layers to classify the healthy and RA patients.

Table 1 depicts the detailed architecture of the RANet model for the categorization of RA and control subjects.

**Table 1:** Detailed architecture of custom RANet model.

Layer	Size	Kernel size	Stride	Activation function	Parameters
Input	256 × 256x3	–	–	–	–
Conv2D_1	256 × 256 × 8	1 × 1	1	ReLU	32
BN_1	256 × 256 × 8	–	–	–	32
Maxpooling_1	128 × 128 × 8	2 × 2	2	–	–
Conv2D_2	126 × 126 × 16	3 × 3	1	ReLU	1168
BN_2	126 × 126 × 16	–	–	–	64
Maxpooling_2	63 × 63 × 16	2 × 2	2	–	–
Conv2D_3	63 × 63 × 32	1 × 1	1	ReLU	544
BN_3	63 × 63 × 32	–	–	–	128
Maxpooling_3	31 × 31 × 32	2 × 2	2	–	–
Conv2D_4	29 × 29 × 64	3 × 3	1	ReLU	18,496
BN_4	29 × 29 × 64	–	–	–	256
Maxpooling_4	14 × 14 × 64	2 × 2	2	–	–
Conv2D_5	14 × 14 × 128	1 × 1	1	ReLU	8320
BN_5	14 × 14 × 128	–	–	–	512
Maxpooling_5	7 × 7 × 128	2 × 2	2	–	–
Conv2D_6	5 × 5 × 256	3 × 3	1	ReLU	295,168
BN_6	5 × 5 × 256	–	–	–	1024
Maxpooling_6	2 × 2 × 256	2 × 2	2	–	–
Global Average Pooling	256	–	–	–	–
FC_1	128	–	–	ReLU	32,896
FC_2	64	–	–	ReLU	8256
FC_3	32	–	–	ReLU	2080
Classification layer (FC_4)	2	–	–	SoftMax	66
Total Parameters – 369,042 Trainable Parameters – 368,034 Non-Trainable Parameters – 1,008					

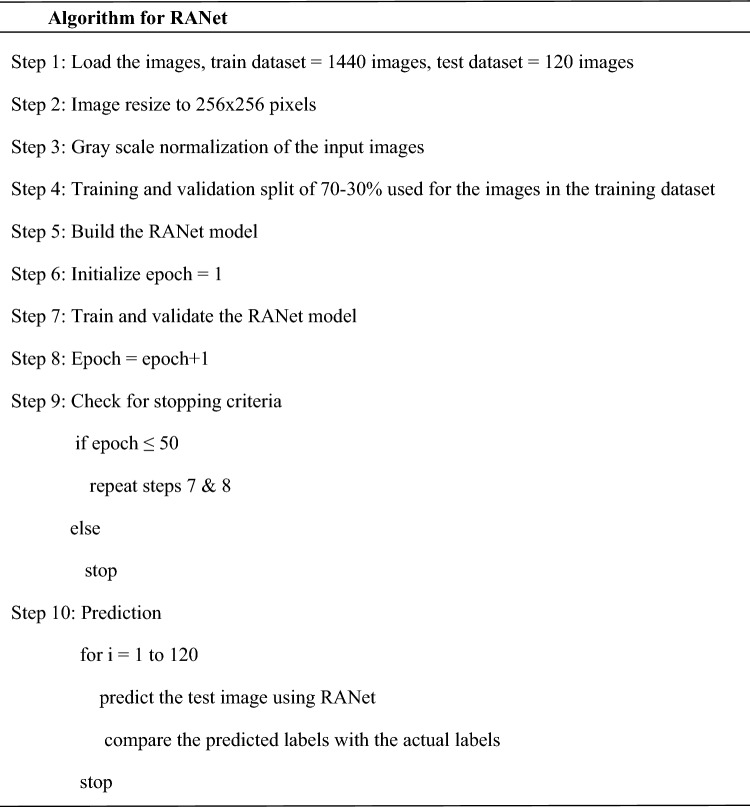



### QNN for the classification of RA

**Figure 6:**
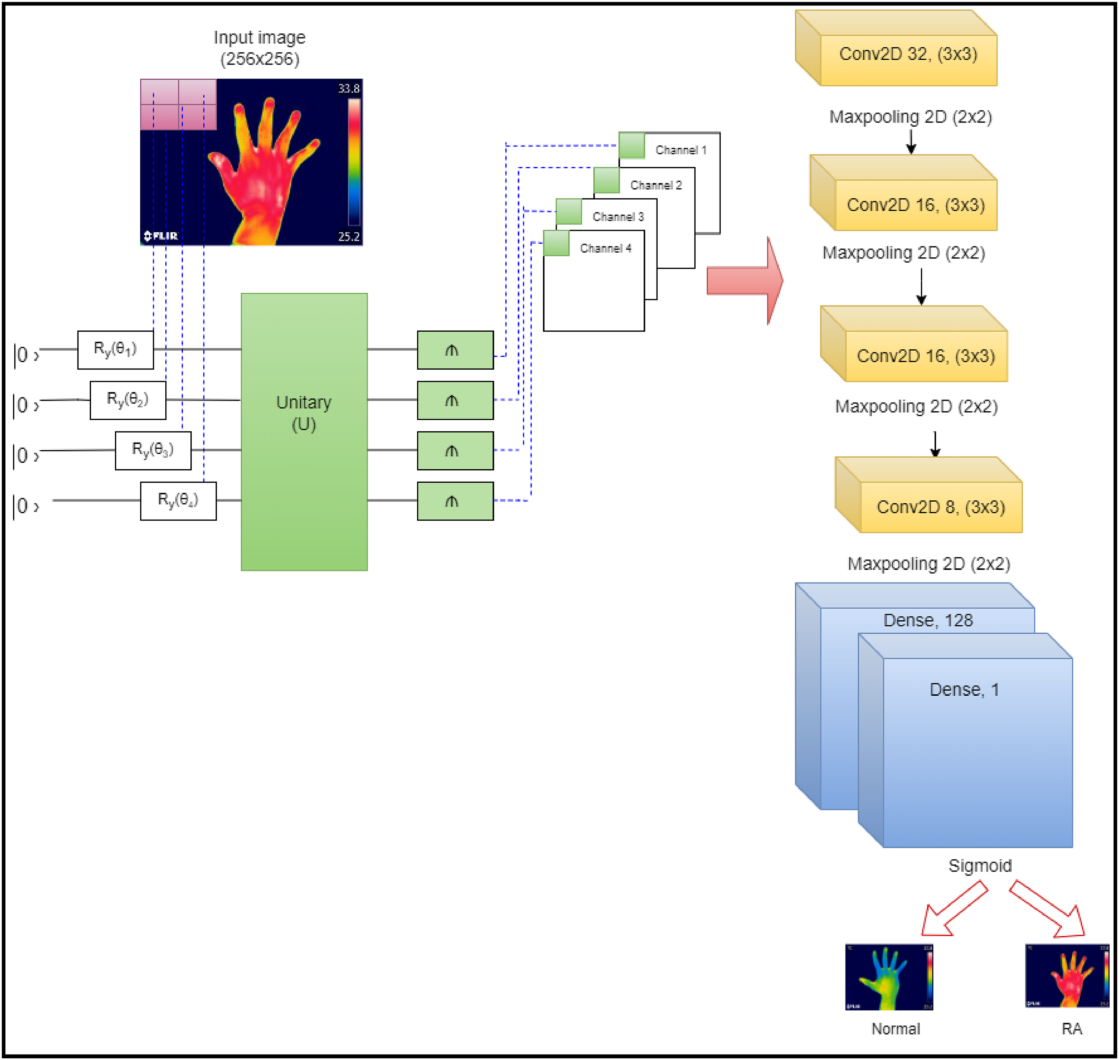
QNN architecture for the classification of RA.

QNN were first proposed by Maxwell et al.^46^ and proved that they could enhance the performance of a classical CNN model. The building blocks of quanvolutional layers are a collection of N quantum filters, which function very similar to their conventional convolutional layer. QNN generates the feature maps by locally modifying the input data. The primary distinction is that quanvolutional filters used random quantum circuits to alter the spatially limited subsets of data to extract the features from the incoming data. For classification purposes, the features generated by random quantum circuits would improve the model performance and accuracy. The proposed study employed four quantum filters or channels, and each channel consists of a unitary random circuit with rotation as the spatial transformation, as shown in Fig. 6. The current study divided the input images of size 256 × 256 pixels into 2 × 2 regions and inserted them into the quantum circuit. The parametrized rotations (Ry), in which a factor of π scaled the rotation angles, were used to generate the quantum images. The study used a quanvolutional layer with four quanvolutional kernels as a pre-processing technique. These quantum-enhanced images were passed into a classical CNN comprised of four convolution layers, 4 max-pooling layers, and two dense layers for the classification of RA as depicted in Fig. 6. The first convolutional layer consists of 32 features of filter size 3 × 3, followed by max pooling layer of size 2 × 2, second and third convolutional layers comprised of 16 feature maps with 3 × 3 filter size preceded by max pooling layer of stride size 2 × 2, and the final convolutional layer consist of 8 feature maps with 3 × 3 filter size. The current study employed two dense layer with neurons 128, and 1 followed by ReLU and sigmoid activation functions. The proposed study used hyperparameters such as learning rate of 0.001, Adam optimizer, binary cross entropy, batch size of 64 and epochs of 30 to train the model.

### Machine learning classification using deep RANet features

In the current study, ML classification was employed to validate the performance of the RANet model by extracting the deep RANet features followed by a feature selection approach. The study employed a tree-based feature selection using a random forest (RF) classifier^47^. The deep RANet features from the third fully connected layer were extracted, and tree-based feature selection using RF was performed. Since the RANet features had labeled targets, we utilized supervised learning ML classifiers such as SVM, k nearest neighbor (*k-*NN), and gradient boosting classifiers for classification. SVM classifier utilizes a hyperplane that effectively classifies the healthy and RA patients^48^. In the SVM model, the hyperparameter 'C' value was set as one and the radial basis function (RBF) kernel function was used. *k-*NN classifier is a supervised algorithm that classifies the features based on similarity^49^. The proposed study used a *k* value of 5 to reduce noise and the effect of outliers in the model. Gradient Boost classifier consist of a group of ML methods that combine numerous weak learning models to create a robust predictive model^50^. In gradient boost classifier, hyperparameters used are 100 estimators, a learning rate as 1, and a maximum depth of 1.

### Ethics approval

The Institute Ethics Committee (Human Studies), SRM MCH&RC, Tamil Nadu, India, IEC Number: 2449/IEC/2021, approved the work described in this manuscript.

### Consent to participate

All participants in the study signed an informed consent form.

## Results

The python programming was run in Google Colab (a cloud-based platform). The entire programming was performed using windows 11 personal computer with 16 GB RAM, 12th Generation Intel^®^ Core™ i7 processor. The training, testing, and validation of the CNN, ML models, and feature selection were executed in Google Colab. QNN classification of RA was performed using ibmq_jakarta, an IBM Quantum Falcon processor based on python programming.

### Categorization of RA patients and control subjects using CNN models

In the proposed work, the CNN models were tested on 120 images after being trained on 1008 images and validated on 432 images. In the proposed study, Stochastic Gradient Descent (SGD) optimizer with a learning rate of 0.01 and batch size of 16 is used in the CNN model. The architecture weights were examined using checkpoints during the CNN model's training. This is determined by the CNN model's weight for classifying the individuals with RA and the healthy participants. The performance measures were evaluated using recall, precision, and F1 measure for the different pre-trained models and RA Net as depicted in Table 2. The RANet model outperformed the pre-trained models with a classification accuracy of 95% as given in Table 2.

**Table 2:** Performance metrics of the various CNN models for RA detection.

CNN model	Class	Precision	Recall	F1-measure	Overall accuracy (%)	Trainable parameters
ResNet101V2	Normal	0.82	0.90	0.86	85	43,094,722
	RA	0.89	0.80	0.84		
	Weighted average	0.85	0.85	0.85		
InceptionResNetV2	Normal	0.78	0.97	0.87	85	54,710,946
	RA	0.96	0.73	0.83		
	Weighted average	0.87	0.85	0.85		
DenseNet201	Normal	0.86	0.93	0.90	89.16	14,887,298
	RA	0.93	0.85	0.89		
	Weighted average	0.89	089	0.89		
RANet	Normal	0.94	0.97	0.95	95	324,736
	RA	0.97	0.93	0.95		
	Weighted average	0.95	0.95	0.95		

**Figure 7:**
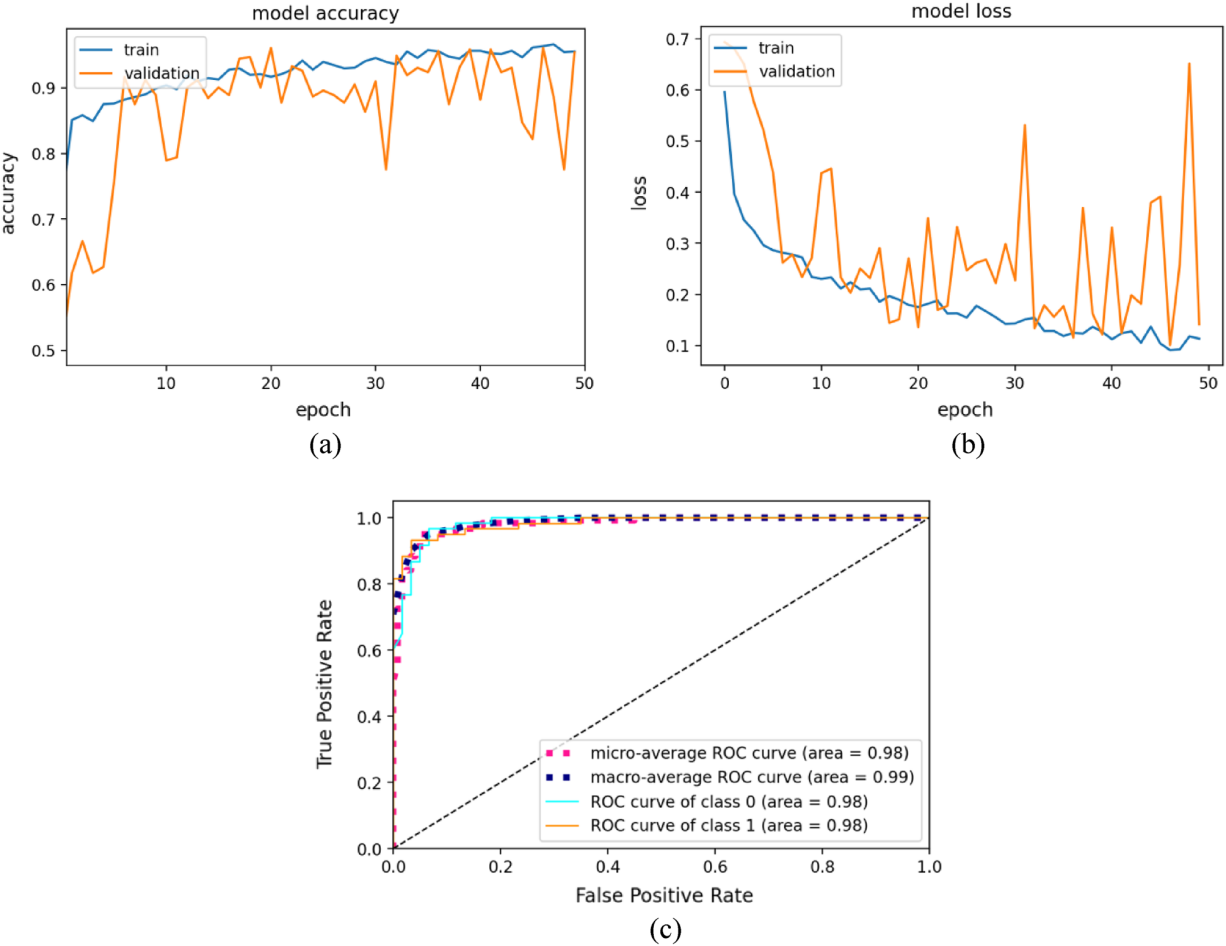
Training and validation plots of the custom RANet model (**a**) classifier accuracy curve (**b**) classifier loss curve (**c**) AUC curve.

Figure 7 illustrates the training and validation plots of the RANet model for 50 epochs. Figure 7a depicts the model accuracy curve of the RANet model, Fig. 7b represents the model loss of the RANet model and Fig. 7c shows the AUC curve of the RANet model. As the number of epochs increases, the accuracy tends to increase and reach a stable state. Similarly, the loss curve tends to decrease, as the number of epochs increases. The AUC value of 0.98 is obtained for RA Net model as given in the ROC graph.

**Figure 8:**
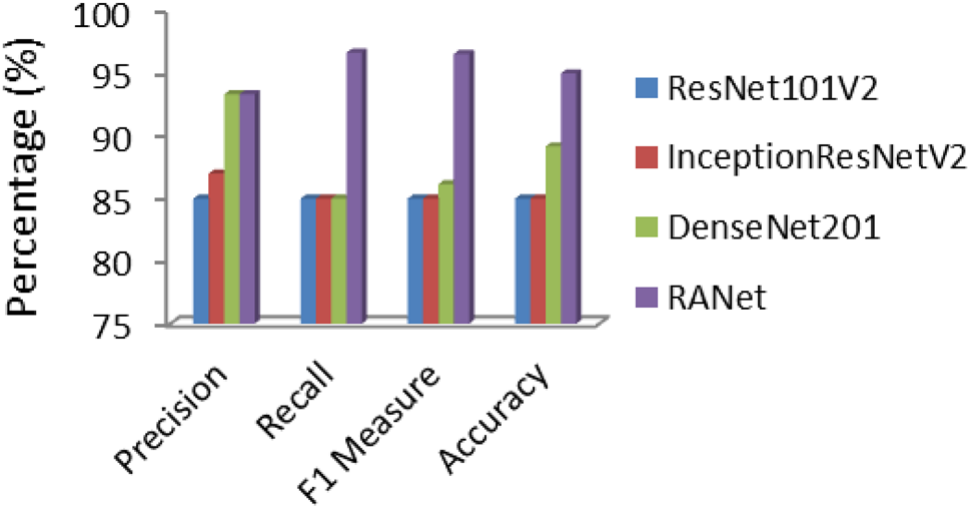
Graphical representation of the performance metrics comparison of the various CNN models.

The graph plot of various CNN models with their precision, recall, F1 measure, and accuracy obtained from the confusion matrix is given in Fig. 8. It was observed that RA Net model outperformed compared to the other pre-trained model in terms all the performance measures.

The confusion matrix in Table 3 depicts the true negative (TN), false positive (FP), false negative (FN), and true positive (TP) for the 120 test images provided by various models utilized in the study.

**Table 3:** Confusion matrix for testing the various CNN models.

CNN model	Performance measures			
	TN	FP	FN	TP
ResNet101V2	48	12	6	54
InceptionResNetV2	44	16	2	58
DenseNet201	51	9	4	56
RANet	58	2	4	56

The comparison based on the training time and error rates of the CNN models are illustrated in the Table 4. For training the RANet model, the system took 107 s and the minimum error rate of 0.05 compared to the pre-trained models.

**Table 4:** Comparison of training time and error rates of the various CNN models.

CNN model	Training time (s)	Error rates
ResNet101V2	579	0.15
InceptionResNetV2	622	0.15
DenseNet201	703	0.10
RANet	107	0.05

### Quanvolutional neural network classification

**Figure 9:**
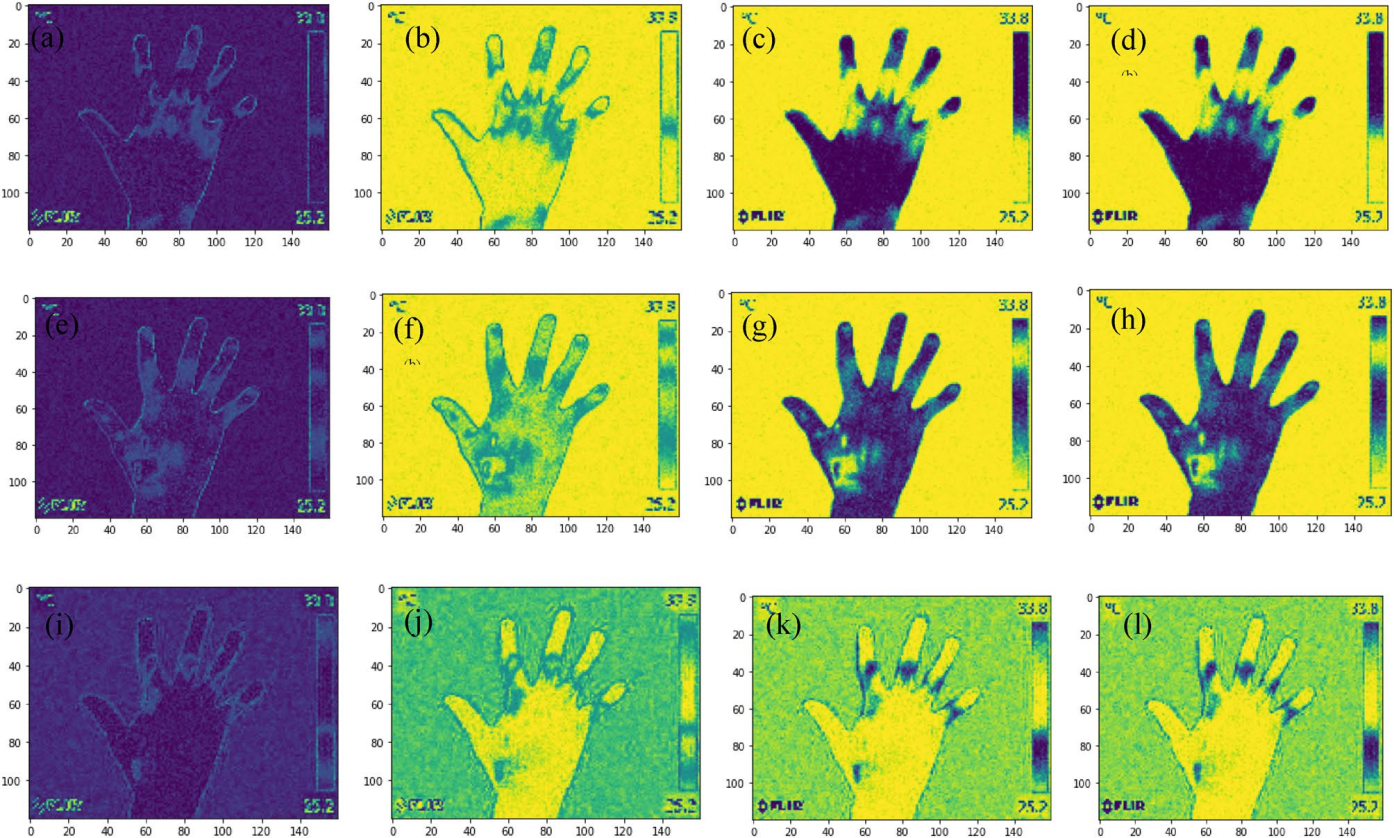
Pre-processed grayscale quantum images generated by the quantum convolutional layer (**a**) Images in Band 1 Quantum channel 1, (**b**) Band 1 Quantum channel 2, (**c**) Band 1 Quantum channel 3, (**d**) Band 1 Quantum channel 4, (**e**) Band 2 Quantum channel 1, (**f**) Band 2 Quantum channel 2, (**g**) Band 2 Quantum channel 3, (**h**) Band 2 Quantum channel 4, (**i**) Band 3 Quantum channel 1, (**j**) Band 3 Quantum channel 2, (**k**) Band 3 Quantum channel 3, (**l**) Band 3 Quantum channel 4.

A total of 1440 images (both control and RA) were used for training and 120 images (normal and RA) for testing the QNN model. Since the 2 × 2 pixel block was embedded in the random circuit, four qubits and a series of rotation gates whose magnitude was based on pixel intensity were used in the quantum circuit. The images got shrunk by a factor of 2 in the x and y direction. The study employed an Adam optimizer with a learning rate of 0.001 and batch size of 64 for training the QNN model. The quantum convolutional layer employed three bands each with four quantum channels in the current work. Figure 9 visualizes the pre-processed quantum images generated by the quantum convolutional layer. From Fig. 9, it is clear that the global shape of the thermal hand image generated by the quanvolutional layer was preserved compared to that of a convolutional layer.

**Figure 10:**
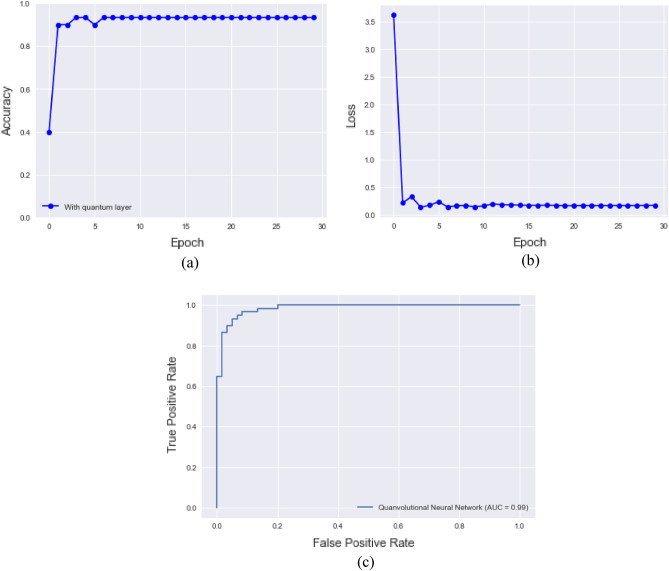
Validation accuracy and ROC curve (**a**) Validation accuracy of QNN model, (**b**) Validation loss of QNN model, (**c**) ROC curve of QNN model.

The QNN model obtained a test accuracy of 93.33% after 30 epochs in the categorization of control and RA subjects. Figure 10 demonstrates the QNN model accuracy, loss plot, and ROC curve of the QNN model.

**Figure 11:**
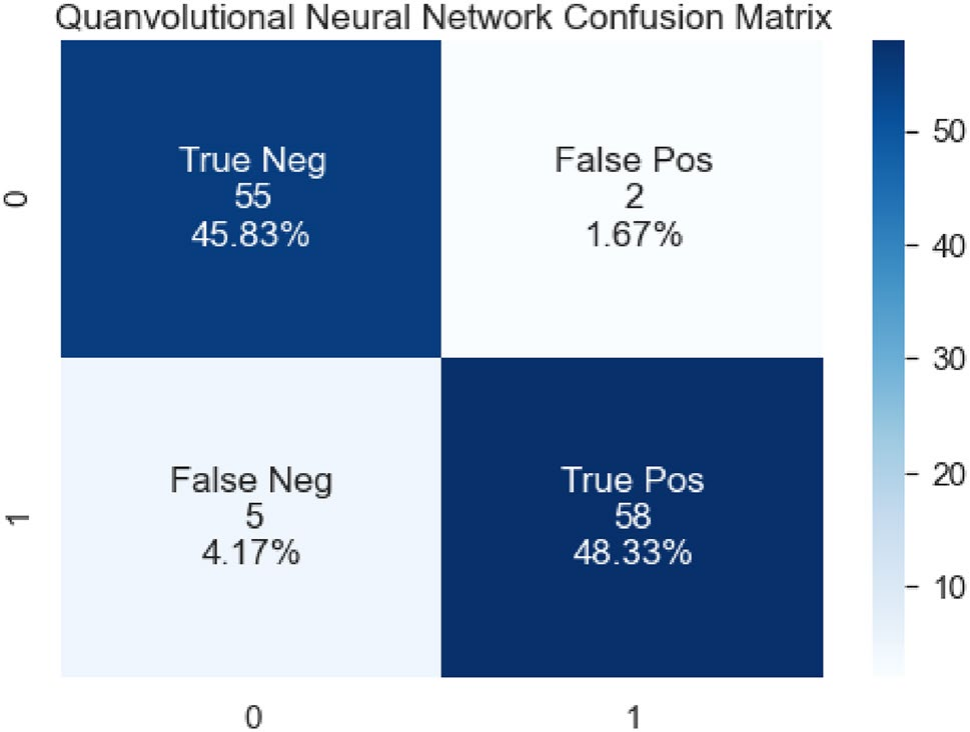
Confusion matrix of the QNN model.

The confusion matrix of the QNN architecture to classify RA and healthy subjects is depicted in Fig. 11. Table 5 represents the performance measures of QNN model which produced a classification accuracy of 93.33%.

**Table 5:** illustrates the performance metrics of the QNN classifier for RA.

Model	Precision	Recall	F1-measure	Accuracy
QNN	0.96	0.92	0.93	93.33%

### Deep RANet feature extraction, feature selection, and ML classification

**Figure 12:**
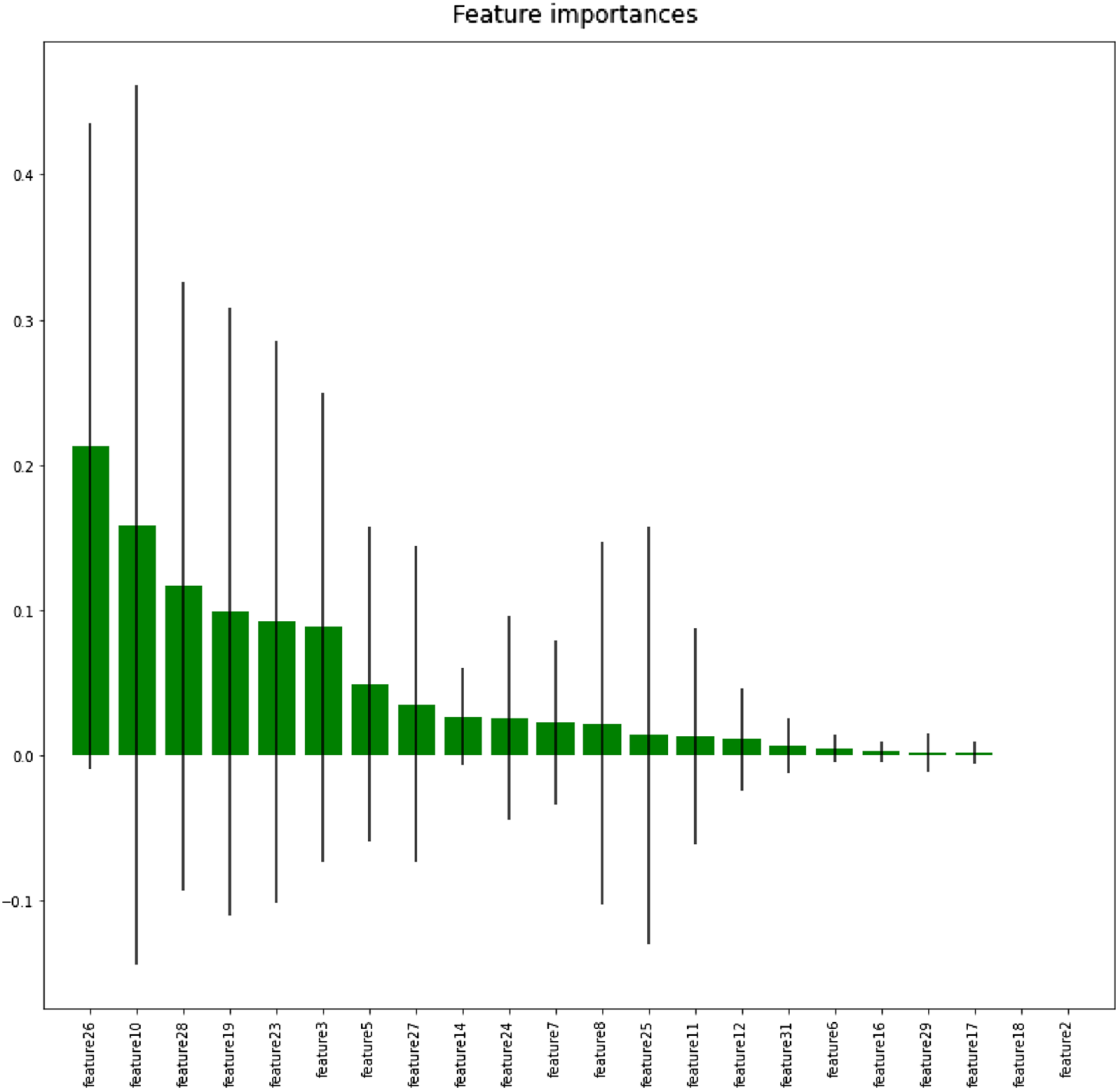
Feature importance based on tree-based RF classifier.

Even though, the custom RANet model provided the highest classification accuracy, this work validated its performance by extracting the deep RANet features, followed by feature selection and ML classification. The deep RANet features were extracted from the third fully linked (connected) layer of the model and fed into the tree-based feature selection using the RF classifier. The features were selected based on the decrease in the mean and standard deviation of the impurity in each tree. The features extracted from the third fully-linked layer of the RANet classifier were divided into 70–30% split for training and testing process. Then feature selection was carried out by using the RF approach. From the deep RANet features, 12 crucial features such as feature 26 (F26), F10, F28, F19, F23, F3, F5, F27, F14, F24, F7, and F8, were selected as exhibited in Fig. 12. The importance of the features decreases after 12 crucial features, as illustrated in Fig. 12.

These features were passed into the ML classifiers such as gradient boosting, *k-*NN, and, SVM models to predict the RA and normal subjects. Table 6 depicts the performance analysis of the various ML models with and without feature selection methods. Among the two ML classifiers, the SVM model with CNN features using the feature selection method outperformed with the highest classification accuracy of 97%.

**Table 6:** Performance analysis of the various ML models with and without feature selection methods.

ML model	With the feature selection method				
	Class	Precision	Recall	F1-measure	Accuracy (%)
Gradient boosting	Normal	0.82	0.86	0.84	80.55
	RA	0.79	0.73	0.76	
	Weighted average	0.80	0.79	0.80	
*k-*NN	Normal	0.95	0.90	0.93	92
	RA	0.88	0.93	0.90	
	Weighted average	0.91	0.91	0.91	
SVM	Normal	1.00	0.96	0.98	97
	RA	0.91	1.00	0.95	
	Weighted average	0.95	0.98	0.96	

ML model	Without a feature selection method				
	Class	Precision	Recall	F1-measure	Accuracy (%)
Gradient boosting	Normal	0.74	0.82	0.78	78
	RA	0.82	0.74	0.78	
	Weighted average	0.78	0.78	0.78	
*k-*NN	Normal	0.81	1.00	0.89	89
	RA	1.00	0.79	0.88	
	Weighted average	0.90	0.89	0.88	
SVM	Normal	0.89	1.00	0.94	94
	RA	1.00	0.90	0.95	
	Weighted average	0.94	0.95	0.94	

**Figure 13:**
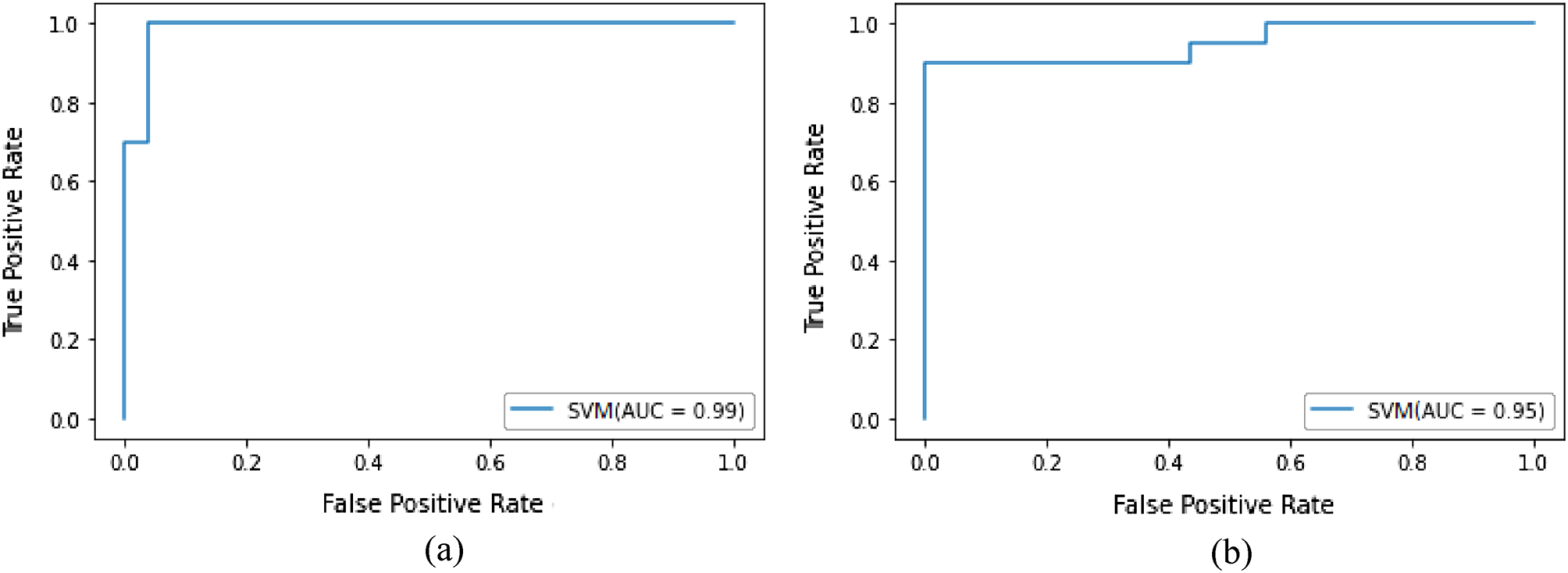
ROC curve of SVM classifier (**a**) with feature selection method (**b**) without feature selection method.

Figure 13 demonstrates ROC curve of SVM classifier with a false positive rate (FPR) on the x-axis and a true positive rate (TPR) on the y-axis. Figure 13a represents the ROC curve obtained after the feature selection method and 13b indicates the ROC curve attained before the feature selection method.

## Discussion

The proposed study used pre-trained models such as ResNet101V2, InceptionResNetV2, and DenseNet201 to classify the RA and healthy controls^51,52^. The performances of these pre-trained models were computationally less effective because it relied on weights from the ImageNet dataset that resulted in negative transfer learning and overfitting^53^. In addition, increased computational time and cost involved in the pre-trained models due to the existence of several trainable parameters and layers. The results showed that the unique RANet model outperformed the pre-trained models. Fewer convolution layers were employed in the RANet, and the activation function like ReLU and batch normalization followed each layer. Since batch normalization streamlines the CNN training process, it reduces the calculation time.

Furthermore, three distinct ML classifiers were used to validate the RANet’s accuracy in the current study. A feature selection method based on tree-based RF was implemented to extract the most relevant features from the RANet model. The performance metrics with and without feature selection methods were compared in which higher accuracy of 97% is achieved in SVM classifier after the feature selection process. The RANet model outperformed the other pre-trained models with accuracy, precision, recall, and an F1 score of 95%.as shown in Table 2. The RANet+ SVM model attained a precision of 95%, recall and F1 score as 98% and 96%, respectively using the tree-based feature selection method using RF.

The elaborate study on RA related literatures were carried out and given in Table 7. The study by Frize et al. detected RA using hand thermograms^54^. The parameters such as mode/max, max–min, and mean/min temperature values were calculated from the finger joints to assess RA. The authors proved that the 2nd and 3rd MCP finger joints showed greater differences in temperature among the normal subjects and RA patients. Their study stated that thermograms could be used as a reliable tool to predict RA. They used limited dataset such as 18 normal and 13 RA patients, hence it is difficult to perform quantitative analysis and decision-making process. Also, they categorized the RA and normal subjects based on the temperature values, which might not be a sufficient parameter for the classification process. A similar study by Snekhalatha et al. performed automated segmentation in hand thermal images to segment the hot spot regions using the *k*-means algorithm^55^. They compared statistical features extracted from the ROI with the skin temperature of healthy and RA groups. Their study proved that the 3rd MCP joint was significantly correlated with the extracted statistical features from the thermal images. The drawback of their study is sample size used was less, and the ML classifiers have not been used in their study for the classification of RA. Pauk et al. assessed RA using demographic, clinical variables and temperature values^25^. The authors performed border dilation and erosion, and extraction of the finger joint is carried out using skeletonization, and object identification is performed using modified first depth search method. They stated that thermography could be used as a pre-screening tool for detecting RA. The limitation of their study includes less sample size and focussed only on temperature parameters measured during cooling and rewarming process. The present study provided a systematic approach by extracting the crucial deep features and automated classification using well-defined pre-trained and custom models. Also, the study proved that the custom RANet model outperformed all other networks. Furthermore, the performance of the RANet model was validated using the various ML classifiers.

**Table 7:** Elaborate survey on literature related to thermal imaging in the evaluation of RA.

Authors	Methods	Input features	Key interpretations
Frize et al.	Manual ROI selection from hand thermal images	Temperature values	MCP 2 and 3 finger joints showed predominant temperature variation between normal and RA subjects
Snekhalatha et al.	*k-*means algorithm for segmentation	Hand thermal images	*k-*means segmented the hot spot regions effectively
Pauk et al.	Normalization, filtration, and skeletonization	Hand thermal images	Thermograms could be used as a pre-screening tool to predict RA
Alarcon et al.	Random forest	Hand thermal images, demographic, and temperature values	Yielded an accuracy of 90% to classify RA using the RF model
Ho et al.	Bagging	Temperature values	Obtained a classification accuracy of 82.5% to predict RA
Ivorra et al.	*k-*NN	Hand thermal images	Attained an accuracy of 79% using the *k-*NN model to classify the RA
Pauk et al.	Artificial neural network (ANN)	Temperature values, demographic, and temperature values	Detected RA with an accuracy of 92.8% using the ANN model
Suma et al.	Manual, color, and *k-*means algorithms	Knee thermal images	*k-*means method found to be an effective technique to segment the hot spot regions
Bardhan et al.	SVM-RFE, RELIEF	Knee thermal images	Obtained an accuracy of 73% for predicting RA using the SVM-RFE method
Kumar et al.	CNN-LSTM	Hand thermal images	Yielded an accuracy of 92.7% to evaluate RA
Naz et al.	Basic CNN model	Hand thermal images	Obtained an accuracy of 66% to assess RA
Proposed method	RANet (custom CNN model)	Features from hand thermal images	RANet attained the highest accuracy of 95% in predicting RA

Alarcon et al. used hand thermal and RGB images, and grip strength as the input features to the various ML classifiers^27^. The authors attained an accuracy of 90% using RF classifier to classify RA in hand thermal images. The main limitation of their study was the hand-crafted feature extraction and selection for the ML classification. The current study employed automated feature extraction using DL models to classify RA. The study by Ho et al. employed an array of temperature values from hand thermal images to predict RA from the control subjects^26^. Their study utilized ensemble ML classifiers such as bagging, random subspace, and AdaBoost with RF and SVM as base classifiers. In their study, AdaBoost yielded the good accuracy of 87.5% with RF as the base classifier to detect RA. The authors suggested that the AdaBoost with base classifier RF could be employed as a screening tool to predict RA. But the authors only utilized temperature data as input to the ML models, ignoring a number of critical factors that define information about RA from hand thermal images. The current study employed intensity-based features related to RA that increases the classification accuracy in the prediction of RA.

Pauk et al. used temperature, demographic, and clinical information as input features to the ANN model^28^. They obtained an accuracy of 92.8% in detecting the RA from the healthy subjects. Their study motivates that the temperature values could be considered a crucial factor reflecting the articular inflammation caused due to RA. But they considered limited parameters such as temperature, demographic, and clinical data as categorizing factors, which is a drawback of their study. Furthermore, clinical indicators such as ESR and CRP will also be high for other diseases, as a result, these indicators cannot be linked to RA symptoms. The intensity variation caused due to RA inflammation was employed in the present study to detect RA. Suma et al. segmented the hot spot regions in the knee thermal images using manual segmentation method, color-based segmentation method, and *k-*means techniques^56^. Their study proved that *k* means clustering algorithm based on the distance of clusters found to be effective in segmenting the hot spot regions from the knee infrared images. The ML classifiers have not been used in their study; instead, they performed the classification based on feature extraction using a computer aided diagnostic tool.

A similar study by Bardhan et al. predicted the subclinical inflammation in knee thermograms using ML classifiers^57^. The authors segmented the ROI using *k-*means, Fuzzy C-means, Otsu, and single-seeded region growing algorithms. Statistical, texture, shape, and frequency level features were extracted from the segmented ROIs. They employed support vector machine-recursive feature elimination (SVM-RFE) and RELIEF models to evaluate the presence of RA in knee infrared images. The authors yielded a better accuracy of 73% using SVM-RFE technique compared to the RELIEF method. Since their study extracted the combination of features, early diagnosis of RA is possible. The segmentation and hand-crafted feature extraction techniques increased the training and classification time which is the drawback of their study. The current study employed DL models, reducing the computational time and cost to classify RA.

Kumar et al. predicted RA using CNN-LSTM (Long short-term memory) technique in infrared images^58^. The authors yielded an accuracy of 92.78% using their model to assess RA. The authors stated that the model could be used as a pre-screening tool to predict the RA due to improved accuracy and precision. The shortcomings of their study are as follows: the combination of CNN-LSTM requires more memory for training the model and is computationally expensive. A similar study by Naz et al. employed a basic CNN model for categorizing the RA from healthy subjects^59^. They attained the lowest accuracy of 66% in classifying the RA and normal participants.

The current study developed a custom RANet model, which proved as an effective diagnostic tool for the classification of RA. Additionally, the custom RA Net model attained the highest accuracy of 95% compared to all other models as discussed in the study. The literature discussed about the implementation of conventional ML and pre-trained models on ImageNet for categorizing the control and RA patients. The proposed study concluded that the custom RANet model could be used as an automated diagnostic tool for detecting RA. Comparing the cost, time consumption, and classification efficiency, the RANet model produced an excellent performance for classifying the RA and healthy participants. Furthermore, the classification efficiency of the RANet model features was validated using distinct ML classifiers, which provided the predominant accuracy of 97%.

The QSVM model^49^ need segmentation and hand-crafted feature extraction methods to classify the RA and healthy subjects. In the case of the QNN model, the hand thermal images were fed into the model without any extensive segmentation and feature extraction techniques. To the best of our knowledge, limited works have been used in the QNN model for RA assessment. This study employed a QNN model to differentiate between the healthy individuals and RA patients. The global hand shape, which had more feature information, was maintained by the feature maps generated by the quantum convolutional layers compared to the CNN architectures. The QNN model distinguished between the RA and control groups, with a test accuracy of 93.33%. Therefore, RA could be evaluated automatically using a computational diagnostic tool such as a customized RANet or QNN model.

### Limitations of the study

The real-time data employed in the current study is limited, and the data were increased using traditional data augmentation techniques. In addition, the resolution of the input images decreased when operating the quantum kernel, and local distortion was obtained. These issues will be overcome only when the QNN simulation model is readily available to implement in a user-friendly platform.

## Conclusions

In conclusion, a QNN and custom RANet model are used for classifying the healthy and RA groups. Firstly, automated feature extraction and classification of the normal and RA were carried out in this work using pre-trained models. A unique CNN model was employed in the research because the existing models fails to produce satisfactory results. Furthermore, this work developed a novel and emerging technology based on quantum computing for the classification purposes. The RANet and QNN architectures achieved the highest accuracy levels, which were 95% and 93.33%, respectively. The RANet model's efficacy was validated by analyzing its features based on feature selection and ML classification. The RANet-SVM model achieved a peak accuracy of 97% using tree-based feature selection with RF, for classifying the healthy and RA participants. Hence, the RANet and QNN models could be deployed as a computer-assisted diagnostic tool for categorizing the RA. Thus, thermal imaging in combination with RANet model could be employed as a pre-screening diagnostic tool to evaluate RA.

## Data Availability

On request from the corresponding author, the data that support the findings of this study are accessible.
